# Engaging With Contemporary Dance: What Can Body Movements Tell us About Audience Responses?

**DOI:** 10.3389/fpsyg.2019.00071

**Published:** 2019-02-01

**Authors:** Lida Theodorou, Patrick G. T. Healey, Fabrizio Smeraldi

**Affiliations:** ^1^Cognitive Science Research Group, School of Electronic Engineering and Computer Science, Queen Mary University of London, London, United Kingdom; ^2^Risk and Information Management, School of Electronic Engineering and Computer Science, Queen Mary University of London, London, United Kingdom

**Keywords:** audience, engagement, motion tracking, movement, contemporary dance

## Abstract

In live performances seated audiences have restricted opportunities for response. Some responses are obvious, such as applause and cheering, but there are also many apparently incidental movements including posture shifts, fixing hair, scratching and adjusting glasses. Do these movements provide clues to people's level of engagement with a performance? Our basic hypothesis is that audience responses are part of a bi-directional system of audience-performer communication. This communication is part of what distinguishes live from recorded performance and underpins live performers' moment-to-moment sense of how well a performance is going. Here we investigate the range of visible real-time movements of audiences in four live contemporary dance performances. Video recordings of performers and audiences were analyzed using computer vision techniques for extracting face, hand and body movement data. The meaning of audience movements were analyzed by comparing clips of the audience at moments of maximum and minimum movement to expert and novice judges. The results show that audience clips with the lowest overall movement are judged as displaying the highest engagement. In addition, we found that while there is no systematic relationship between audience and dancers movement, hands seem to play an especially significant role since they move significantly more compared to the rest of the body. We draw on these findings to argue that collective stillness is an especially salient signal of audience engagement.

## 1. Introduction

In many live performances, audiences are separated from performers; seated in the dark observing the performance. The primary conventional opportunity for members of an audience to express their satisfaction or dissatisfaction with a performance is through applause and/or cheering. Nonetheless, audiences have notoriously recruited other means of signaling their responses including the organized and carefully timed use of apparently innocent activities such as coughing (Broth, 2011; Wagener, 2012).

Our programmatic hypothesis is that audience responses are part of a bi-directional system of real-time audience-performer feedback that distinguishes live from recorded performance. A key motivation for this hypothesis is that performers routinely distinguish between “good” or “bad” audiences for the same performance and between specific moments of audience engagement or “lift” and moments of disengagement or boredom (Healey et al., 2009). This raises the question of what performers are detecting in these situations that feeds their ongoing sense of how engaged the audience is during a performance. In cases such as stand-up comedy, the ongoing feedback between audience and performers can be especially obvious e.g., the use of shouting, laughter and heckles. Here, we consider a much more challenging case; contemporary dance. Dance has quite different conventions about what forms of audience response are considered appropriate; laughter is rare and shouting and heckling are definitely out. In a typical contemporary dance performance the audience will be in the dark, the performers behind bright lights with loud music, drowning out other sounds. There are few, if any, opportunities for direct eye contact or verbal exchanges between performers and the audience. In addition, dancers need to contend with the physical and cognitive demands of the dance performance itself. This places severe limits on what dancers are able to sense, even in principle, about audience responses during a performance. Almost the only available channel of communication between audience and performers is body movements.

One hypothesis about the possible connections between audiences and performers during a dance performance is kinesthetic empathy (Reason and Reynolds, 2007; Winters, 2008; Jola et al., 2011a). According to Calvo-Merino et al. (2004) affective responses to body movement can be explained in terms of “kinesthetic” proprioception. Reason and Reynolds (2007) proposes that the ideal spectators in a dance performance are those that use this response to become participants in the movement that is presented to them by recreating the dance movements with their own musculature. To the extent that this simulative process produces perceptible body movements it provides a potential channel for communication between dancers and their audiences. However, in most of the studies that have tested kinesthesia the activity of the spectators is measured indirectly (using fMRI or TMS) in laboratory based experimental conditions which substantially impede the live experience.

Previous work on live dance audiences has revealed a relatively rich repertoire of different audience body movements: scratching, adjusting hair, adjusting glasses, supporting the chin and drinking amongst others (Theodorou et al., 2016). These movements are not obviously connected with the movements of dancers but are nonetheless at least partially visible to them. Do these movement provide a signal of audience engagement and thereby form part of a feedback cycle between the performers and their audience?

## 2. Literature Review

### 2.1. Live Audience Response Metrics

Finding ways to measure moment-by-moment audience engagement in real theater settings is essential for getting a better understanding of the live experience. It also has the potential to enable new forms of creative production. While live audience response metrics can be used as a way of analyzing or “debugging” a performance, they can also enable new forms of dynamically responsive, creative intervention.

Performance unfolds in time, making data collection problematic (Schubert et al., 2009). A growing number of studies in dance research use motion sensing technologies but primarily to examine dance movements (Camurri et al., 2003; Calvo-Merino et al., 2004; Leman and Naveda, 2010). In contrast to this very little research has focused on audiences (for exceptions see e.g., Healey et al. 2009; Stevens et al. 2009; Vincs et al. 2010; Gardair et al. 2011; Jola et al. 2011b; Latulipe et al. 2011; Mann et al. 2013; Katevas et al. 2015; Theodorou et al. 2016; Vicary et al. 2017). There are many possible ways to measure audience engagement in the performing arts. The most common approaches involve the use of post-performance questionnaires, focus groups and audience interviews (Stevens et al., 2009; Pasquier, 2015). These are useful for investigating paticipants' narratives and interpretations of a performance however, they also have the disadvantage of being essentially retrospective. This can lead to problems such as the “peak-end” effect, which shows that a measure taken immediately after an experience is strongly influenced by the emotion experienced at the end of the performance (Latulipe et al., 2011). In order to address the dynamic experience of the performing arts, real-time measures of response are needed rather than discrete, post-performance measures to capture audience engagement (Schubert et al., 2009). This enables fine-grained quantitative analysis, offering a new perspective on the dynamics of audience responses.

A variety of quantitative measures of audience engagement have been tried, which can be divided into overt responses that are expressed through visible human actions, movements or expressions and covert responses, that are manifest in biochemical and electrical changes of the human body. Overt measurements include facial expressions (Katevas et al., 2015; Theodorou et al., 2016), body movement (Healey et al., 2009; Gardair et al., 2011; Theodorou et al., 2016; Vicary et al., 2017), eye movements (Stevens et al., 2009) and continuous self-rated measurements (McAdams et al., 2004; Vincs et al., 2010; Vicary et al., 2017) while some examples of covert responses that have been used are brain activity (Calvo-Merino et al., 2004; Jola et al., 2011b), galvanic skin response (GSR) (Latulipe et al., 2011; Wang et al., 2014), heart rate variability (Shoda et al., 2016; Vicary et al., 2017). To the extent that these covert responses are “invisible” to performers they cannot form the basis of an ongoing performer-audience feedback loop.

### 2.2. Non Verbal (Visible) Cues of Boredom and Engagement

There is currently no accepted theory of what audience engagement and/or boredom are, partly because of conflicting definitions. However, in this section we will briefly discuss some definitions coming from literature from performing arts (Stevens et al., 2009; Vincs et al., 2010; Latulipe et al., 2011), psychology (Kahn, 1990; Csikszentmihalyi, 1997; Macey and Schneider, 2008) and Human Computer Interaction (HCI) (Chapman and Webster, 1999; Bianchi-Berthouze et al., 2007; Brien and Toms, 2008; Witchel et al., 2014). Existing research suggests that both boredom and engagement are associated with specific body postures, including the position of the head (Bull, 1978; D'Mello et al., 2007; Witchel et al., 2014) torso (Grafsgaard et al., 2013) and hands (Grafsgaard et al., 2013). For example, according to Bull (1978) there are specific head positions that characterize boredom such as “drops head,” “turns head,” and “head lean.” However, Witchel et al. (2014) argue that body posture alone is not a sufficient marker of engagement and also depends on the kind of stimulus and interaction needed. Apart from body posture, body speed is another measurement that has been used to identify engagement and boredom, particularly for games or tutorial systems where interaction rate was controlled by the user. HCI research suggests that the increase of overall body movement is related to boredom and frustration while diminished movement is related to engagement (D'Mello et al., 2007; Kapoor et al., 2007; Grafsgaard et al., 2013).

One issue for the definition of engagement and/or boredom in passive tasks like watching television or attending a performance is that they can be performed in high or a low activity states. Restless activity includes fidgeting or stunted escape efforts while lethargic boredom might manifest itself in the viewer resting their head on their hand with elbow support (load bearing). A similar argument holds for engagement: dynamic engagement could be a football fan raising their arms in celebration of a goal, while rapt engagement might be a child watching a cartoon in perfect stillness (Witchel et al., 2014). This suggests that in relatively passive tasks like the one we study here it is not straightforward to distinguish between engagement and boredom.

Previous research (Theodorou et al., 2016; Theodorou and Healey, 2017) has shown that body fidgeting and self touching gestures (STGs) are relatively frequent in audiences and also potentially detectable by performers. Fidgeting is commonly defined as a general indication of boredom, irritation, and lack of attentional engagement. In an early test of this claim, Galton (1885) observed fidgeting behaviors of audience members during a boring lecture. Galton (1885) observed that when the audience was more engaged the frequency of fidgeting reduced by more than half and the duration of each movement also reduced. According to Galton (1885):

“When the audience is intent each person forgets his muscular weariness and skin discomfort, and he holds himself rigidly in the best position for seeing and hearing. But when the audience is bored the several individuals cease to forget themselves and they begin to pay much attention to the discomforts attendant on sitting long in the same position. They sway from side to side, each in his own way and the intervals between their faces which lie at the free end of the radius formed by their bodies with their seats as the center of rotation varies greatly.”

Similarly, according to a 68 year-old theatergoer interviewed by Pasquier (2015), audiences' increase of body movement shows disengagement:

“When one's concentration goes, the body needs a release, by crossing one's legs, sitting up on one's chair…and coughing of course. That's the cacophony of failure. One senses the dispersion, people who start moving, changing position, who're leaning like this on their hand, who dip their head or look at others, you feel they're thinking 'shit, this is never going to end', who look at their watch, so it does show. I've got antennae…” (Pasquier, 2015).

An obvious candidate visual signal of fidgeting during a performance is hand movement. Theodorou and Healey (2017) showed that audiences have their hands on their faces for about half a dance performance and that the hands move faster when they are up compared to when they are down in a resting position. Despite their visibility, these movements are not typically thought of as communicative. According to Harrigan et al. (1987) STGs lack overt, intentional design and may be performed with little or no awareness.

There is evidence of an increase in self-touching behavior in stressful and fearful situations. Butzen et al. (2005) found a significant increase of STGs in response to a video about chiggers compared to a less disturbing video. In a study from Heaven and McBrayer (2000) participants listened to texts about leeches and canaries and then had to answer several questions. Although there were no differences between the two listening conditions there was an increase in STGs for the leeches text during the answering period. Rogels et al. (1990) found that children between 3 and 6 years showed more self-touch gestures while talking about a cartoon they had just seen than while watching the cartoon. Other studies (Grunwald et al., 2014) hypothesize that there is a relationship between the frequency of STGs and arousal. Barroso and Feld (1986) investigated this by testing the occurrence of self-touch gestures performed with one or both hands as a function of four different auditory attention tasks. They found that with increasing complexity and attentional demands both one and two handed self-touch gestures increased. A recent study of different categories of hand-over-face gestures included possible interpretations ranging over cognitive, affective states such as thinking, frustration, or boredom (Mahmoud and Robinson, 2011). Ekman and Friesen (1972) has suggested that STGs may also occur when a person is relaxed.

In summary, the claims in the literature about the relation between body moments and engagement or boredom are not entirely consistent and seem to depend on the social context of the activity. Based on our previous studies and on the literature presented above we believe that in the context of contemporary dance audience body movement might give us information about audience engagement to the performance. In particular, hand movements are, in principle, visible to performers and there is evidence that they are systematically connected to people's level of interest in what is happening around them. In contrast to the kinesthetia hypothesis, our proposal is that audience body movements in general, and hand movements in particular, are broadly symptomatic of audience disengagement. Note, we are interested here in the ongoing movements of the audience *during* the performance not the conventional responses, such as applause, at the end of a piece. This leads us to three basic hypotheses:

***Hypothesis 1 (H1):*** *Audience hand movements provide a specific, distinct and salient response cue to performers*.

Hypotheses 2 and 3 examine the relationship of body speed with engagement and/or boredom and test the kinesthesia hypothesis described in the introduction:

***Hypothesis 2 (H2):*** *Movement and engagement are inversely correlated*.

***Hypothesis 3 (H3):*** *Audience movement can be predicted from dancers movement*.

We investigate this by first mapping the general face, body and hand behavior patterns displayed by an audience and then focus on the potential relationship between engagement and body movement.

## 3. Materials and Methods

In order to test the hypotheses described above we primarily relied on continuous audience measurements collected in a real theater setting. Collecting continuous audience and dancers data in real theatrical settings and not in a laboratory was one of the main priorities of this research. This decision is motivated by the notion that the social behavior of audiences and dancers will be influenced by the environment. Removing dancers and audiences from their “natural” environment might lead to changes in their behaviour. Social actions and identities are contextual and transferring participants to a laboratory to make a controlled study removes this context. In addition to that, self-reported data was collected using two online surveys, one as a proxy for identify how engaging the performance was and one to determine how engaged the audience was.

### 3.1. Performances

**Figure 1:**
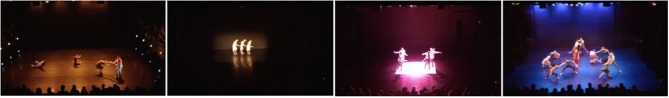
Performance Parts 1–4 (from left to right) performed by LCD.

The collection of the data took place at “The Place” theater in London where four contemporary dance pieces performed by dancers of the London Contemporary Dance School (LCD). The performance lasted for 1 h and 40 min and consisted of 420 min dance pieces (see Figure 1). There was a 15 min interval between the second and the third piece and two 3 min interludes after the first and the third piece. Each dance was performed by LCD postgraduate students and directed by commissioned professional choreographers. The first piece, “Les femmes meurent deux fois” was directed by the choreographer Danae Morfoniou. This piece starts with a pre-performance part during which the lights are turned off, the music starts but there are no dancers on stage. When the music stops, the dancers appear on stage and start performing the first choreographic part without the accompaniment of music. The second piece “Triptych,” was directed by Mara Vivas. This is the quietest among the four pieces since for the majority time the dancers perform synchronized, gentle movements in silence. The third performance is called “The Endgame” and was directed by the choreographer Olatz de Andres. In comparison to the other three, this piece includes different theatrical effects and many artistic changes (lighting and music changes). The fourth performance,“The Tide” was directed by Tom Roden. In addition to the dancing part, this piece also includes some acting parts. There is no dialogue among the dancers but a narrator is on stage during most of the performance. As part of our study on audience responses, we filmed audiences and dancers during the four parts of the performance.

### 3.2. Equipment Set-Up

**Figure 2:**
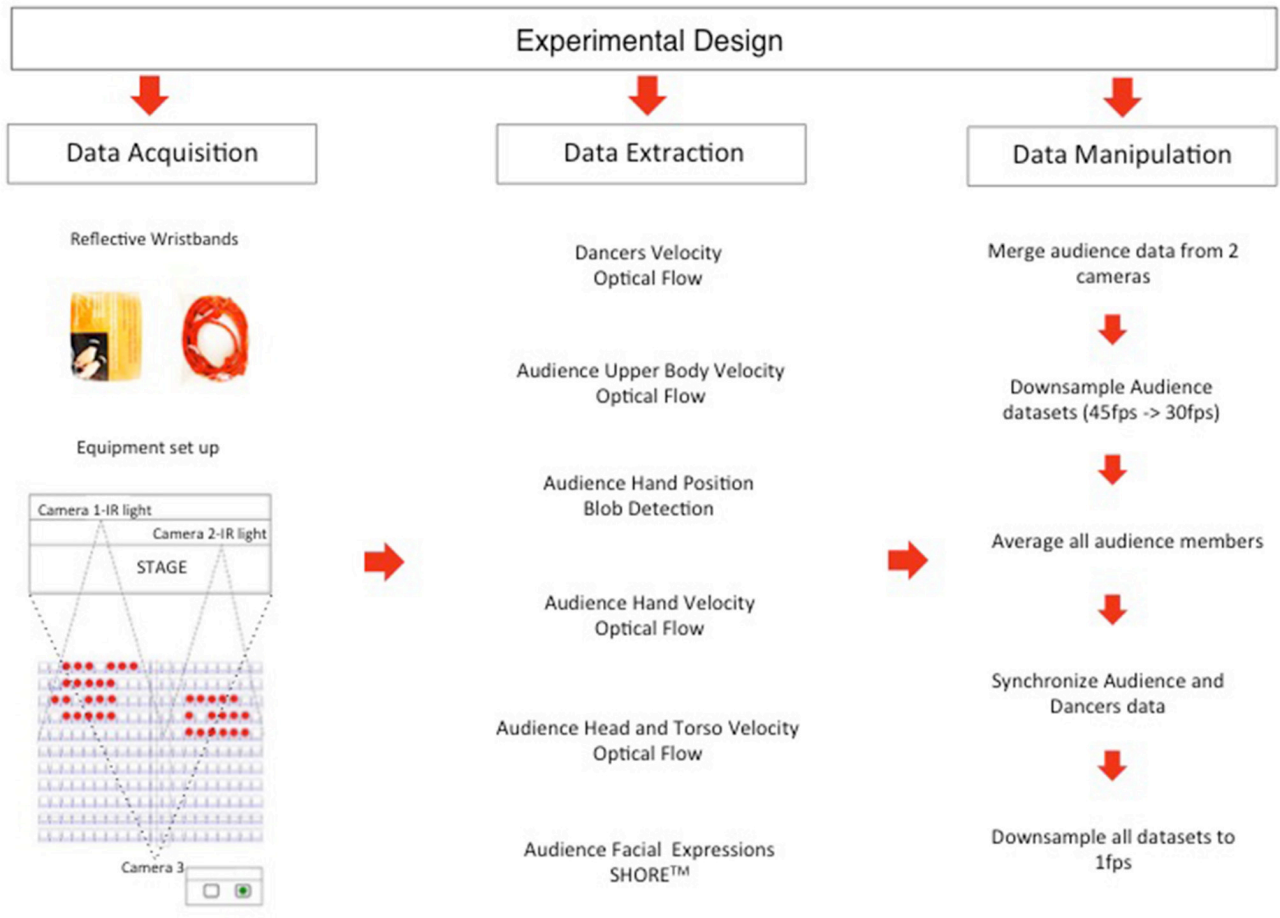
Data processing pipeline.

In order to be able to capture a big enough sample of the audience, we used two Basler Ace (1,280 × 1,024 px resolution) night vision cameras (45 fps). An infrared light (IR) was attached on top of each camera to allow us to film the audience during the dark periods of the performance. Both cameras and IR lights were placed on the theater truss on top of the stage pointing toward the part of the audience to be filmed (see Figure 2). We also filmed the dancers using a JVC professional camera (29.97 ps) which was hung from the rig facing the stage. For the synchronized double GEV camera recording we used the Gecko software made by Vision Experts. Gecko gave us better data accuracy since the video recording used a fixed framerate with a timestamp on each frame. This helped to avoid any synchronization problems by automatically synchronizing the two cameras that were filming the audience but also enabling a more accurate synchronization of the recordings of the audience and the performance. In addition to filming the audience and the dancers, we aimed to track the hand (wrist) movements of each audience member automatically. In order to do this, we created wristbands made of 5 mm reflective rope. A small plastic bag with two reflective wristbands together with instructions on how to wear them was placed on the arm of each theater seat (see Figure 2). Each audience member had to wear one wristband on each hand. As the IR lights were facing directly on the audience, the wristbands became very visible in the video recordings. We researched and identified multiple solutions to automatically track and record continuous wrist movements and this solution was the cheapest and easiest for our available budget and time. Privacy was also an issue in this study since we aimed to extract personal data from the audience members. The study was certified with an ethical approval from the Ethics Committee of Queen Mary University of London (Ethical approval reference number: QMERC1432a) and a sign was placed on each seat to inform audience members that filming was taking place during the performances for research purposes.

### 3.3. Continuous Data

To obtain fine-grained response measures from the footage of the audience and dancers we used data analysis techniques developed in computer vision research. The data processing pipeline (see Figure 2) consisted of: (1) Blob detection algorithm from the Blobscanner Processing library (Molinaro, 2010) used to detect and extract the continuous position of the wrist of each audience member (2) Optical flow algorithm made by Borenstein (2013) in Processing used to calculate the visual change in both the footage of the audience and the dancers (3) SHORE^*TM*^ a facial analysis software made by Fraunhofer Institute (Küblbeck and Ernst, 2006) for Integrated Circuits used to extract all the facial expressions of each audience member during the performance.

#### 3.3.1. Visual Change in Dancers' Videos

Apparent visual motion in the performance videos was measured using the optical flow algorithm. Optical flow estimates frame-to-frame motion by measuring the flow of gray values on the image plane. Under reasonable assumptions, this approximates the projection of the actual motion field in the 3D scene over the camera plane (Jähne, 2005). A number of optical flow algorithms are available in the literature. We used the robust algorithm presented in (Farnebäck, 2003), that is well suited to the challenging illumination conditions of our study; specifically, we relied on the openCV for Processing implementation made available by Borenstein (Borenstein, 2013). For the purpose of our study, we considered the integral of the magnitude of the flow field across the entire frames of the dancers' videos. This represents an estimate of the average level of motion of the dancers; high values result from either fast motion in one area of the stage, or distributed motion across the scene, irrespective of the direction of motion and of its coherence. In the rest of this paper, we will refer to this as the “average speed.”

#### 3.3.2. Audience Upper Body Movement

Optical flow was also used to estimate the average upper body speed of each audience member separately. This included the head, the torso and the hands. Specifically, a static polygonal envelope was drawn around each audience member and the magnitude of optical flow was integrated over each of these envelopes. This method is based on the assumption that during a performance seated audiences are only able to move in a limited area; motion outside the envelope would not contribute to the integral. This can reduce the accuracy of the results in some cases (see section 4.2 for more details).

#### 3.3.3. Audience Hand Movement

For the detection of hand motion we relied on the wristbands, and used the blob detection algorithm provided by the Blobscanner library for Processing (Molinaro, 2010). The algorithm is based on connected component detection and brightness thresholding; the threshold was set manually based on the observation that the reflective wristbands stand out in the images as regions of high intensity under infrared illumination. By applying this method to each frame we extracted the image coordinates of all the wristbands, which allowed us to track the right and left wrist positions of audience members.

Due to pose changes and self occlusions completely automated tracking throughout the performance was unreliable. We therefore used the algorithm to obtain an initial set of traces that were subsequently overlaid on the footage of the entire performance and corrected or disambiguated manually as required. In order to maintain coherence with section 3.3.2 and section 3.3.4 below and also to capture information from the hands properly, we chose not to differentiate the coordinates of the wristbands directly. Instead, we used the continuous position of the wristbands to anchor a rectangular neighborhood covering the region of each hand. We then proceeded to integrate the magnitude of the optical flow field (section 3.3.1) frame by frame over these hand regions to obtain an estimate of the average speed of the hands.

#### 3.3.4. Audience Head and Torso Movement

In order to be able to test the significance of the hands in the performance, we compared their behavior with that of the rest of the body. To isolate the head and torso movement of the audience we integrated, for each person, the magnitude of the optical flow field over the polygonal envelope defined in section 3.3.2 minus the hand regions identified in section 3.3.3 above and applied optical flow. Note that this is not equivalent to a simple difference of the timeseries computed in sections 3.3.2 and 3.3.3, as the hand regions may or may not overlap with the static envelope. This procedure gave us an estimate of the upper-body movement of each person excluding hand movements.

#### 3.3.5. Audience Facial Expressions

A computer vision framework, SHORE^*TM*^ (Sophisticated High-speed Object Recognition Engine) (Küblbeck and Ernst, 2006) was used to extract continuous measures of the degree of displayed happiness, sadness, surprise and anger for each audience member described as percentages. SHORE^*TM*^ is a cross-platform computer vision framework designed by the Fraunhofer Institute for Integrated Circuits for detecting, analyzing and identifying faces from video streams. The happiness analyser has been validated on the JAFFE data base (95.3% recognition rate) while the other three are unreported. Further information can be found on the Fraunhofer IIS website: http://iis.fraunhofer.de. SHORE^*TM*^ was able to accurately detect faces in only one of the two video recordings due to high tilt angle of the camera in the second one. For an accurate tracking SHORE^*TM*^ requires a minimum face size in the image of 35 × 35 px. This requirement was covered in our video recording. However, it should be noted that these estimates are not always reliable as that there are short video segments in which the software was not able to detect enough faces primarily due to the rotation of the head or people placing their hands on their face. Nonetheless, based on other researchers (Katevas et al., 2015) that used the software in similar conditions the measure appears to be robust over extended periods.

#### 3.3.6. Data Preprocessing

We used the VirtualDub software application to downsample the videos from 45 to 30 fps, in order to synchronize the audience recordings with that of the performance. ELAN, a professional tool for the creation of complex annotations on video resources, was used to synchronize the three videos together (two videos of audiences and one of dancers). For data analysis purposes we merged the audience data from both cameras in one data set. In total we have 48 audience members from the two video recordings. However, the sample size of each data set varied depending on the tracking method used to export the data (see results section for details). In summary, we calculated six timeseries variables for each performance part. One was extracted from the performers: the visual change that was produced on screen (described in section 3.2.1). Five timeseries variables were derived by averaging the spectators following datasets: facial expressions (displayed anger and happiness), speed of the hands, head and torso and total upper body. It was decided that a sampling rate of 1 Hz for the compiled data set was appropriate given earlier studies (Schubert, 2004), which indicate that real-time perceptual responses generally take at least 1–5 s for full registration.

### 3.4. Self-Reported Data

In order to test whether less movement in the audience correlates with more engagement in the performance (H2) we relied on self-reported metrics collected using two online surveys. The first survey was used to collect information about the four performances and the second focused on the evaluation of selected audience responses. Due to the difficulty of acquiring any information from the audience members that we filmed the day of the performance, video recordings of the performance and the audience were used to collect data from different audience samples.

#### 3.4.1. Survey I: Ranking the Performances

The main aim of the performance survey was to identify any global differences in participants' preference for the four performance parts. The survey consisted of five questions and was sent to 22 participants (3 males). The age groups were 18–29 (9 participants), 30–39 (6 participants), 40–49 (1 participant), 50–59 (3 participants), and over 60 (3 participants). Thirteen participants reported they like to watch dance as spectators, while the other 9 had some sort of professional connection to dance (e.g., dancer, actor/actress, musician etc.) The main question of the survey asked the participants to watch the video recording of each performance part and then put the parts in an order of preference from 1 to 4, where 1 is the most preferable and 4 the least. The order of the performances on the form was different for each participant.

#### 3.4.2. Survey II: Assessing Audience Engagement From Movement

The second survey focused on participant's rating of audience engagement to the performance by watching short selected clips showing the audience. The survey consisted of 2 sections and was sent to 13 participants (5 males). The age groups were 18–29 (4 participants), 30–39 (4 participants), 40–49 (4 participants), and 50–59 (1 participant). Eight of the participants reported that they like to watch dance as spectators while 5 of them were professionally connected to dance.

The main section of the survey included the audience clips from each performance piece. The clips selection was made based on the upper body movement data. Looking at the upper body movement timeseries of the audience from one of the two cameras, six short clips were selected showing the audience for each of the four performance parts (24 clips in total since there were 4 performance parts). The clips were added to the online survey accompanied by the following question: “On a scale of 0 to 10, how engaged is the audience in the video below? (0 = Not at all Engaged and 10 = Very Engaged).” Under each clip there was a slider with values from 0 to 10. The order of the clips on the form was different for each participant. See Supplementary Material for an example of two of the selected clips.

## 4. Results

Results are reported in three parts. Firstly, we examine the audience responses separately for facial expressions, head/torso and hand movement to test hypothesis 1. Then, we compare the continuous audience responses with the subjective responses collected from the survey to test the key hypothesis that less movement in the audience is associated with moments of audience engagement (H2). Finally, we test the kinesthesia hypothesis (H3) by examining the relationship between audience and performer movements. Our key findings are summarized in the discussion section.

### 4.1. Facial Expressions

Facial tracking was applied on one of the two audience video recordings using the SHORE^*TM*^ software which was able to track on average 10 out of 17 faces. The software managed to reliably track the same persons during the duration of the recording, with minimum number of persons tracked 5 and maximum 17. As expected, tracking was least reliable during the interludes where audience members move more. The measures of displayed happiness, anger, surprise, and sadness produced by SHORE^*TM*^ showed substantial inter-correlations. For example, happiness and anger levels are negatively correlated (*r* = –0.44, *p* < 0.001).

**Figure 3:**
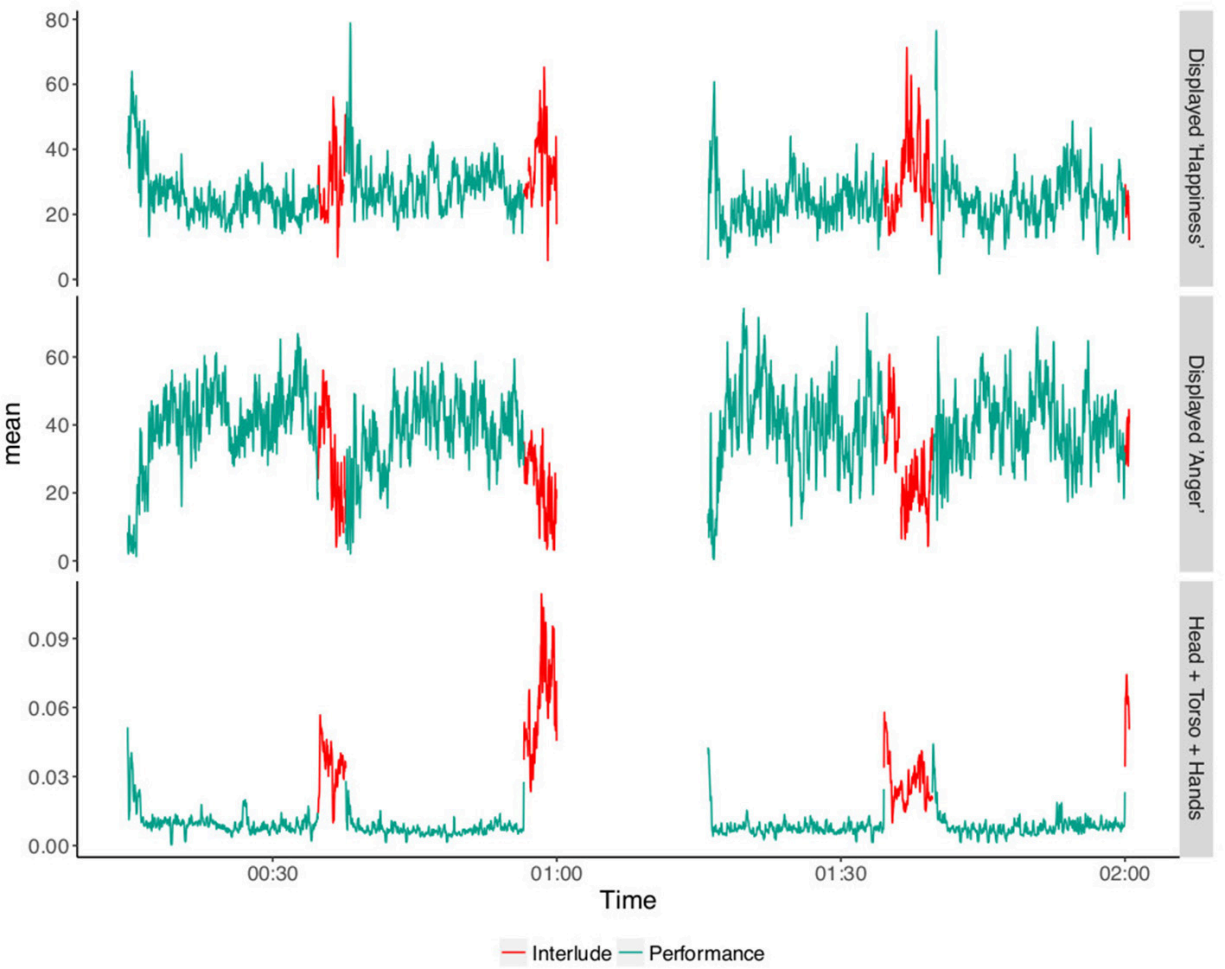
Continuous responses during performance and interlude parts averaged across participants. X axis shows the time in hours and Y axis the average values for each measurement.

The top two line plots in Figure 3 show the average levels of displayed “happiness” and “anger” during the performance parts and during the non performance parts (including the applause sections). Both average happiness and anger displayed by the audience were analyzed in a Generalized Linear Mixed Model (GLLM) using a Linear Model. For this the performance state (Non-performance or Performance) was defined as a fixed factor and audience member as a random factor.

The results of the model show a main effect of performance state in audience displayed happiness (Chi-sq = 109.22 , *p* <0.01) and on displayed anger (Chi-sq = 300.3, *p* < 0.01). The GLMM results are reported in Tables 1, 2 below.

**Table 1:** GLMM model for displayed “happiness” (performance vs. non performance).

	**Estimate**	**Std. error**	**df**	***t*-value**
During performance (happy)	–2.05	0.20	29255.96	–10.45

**Table 2:** GLMM model for displayed “anger” (performance vs non performance).

	**Estimate**	**Std. error**	**df**	***t*-value**
During performance (angry)	5.04	0.29	29257.89	17.33

It is important to note that the SHORE^*TM*^ measure of displayed “anger” does not, in this context, correspond to actual anger but rather signals a blank or relatively expressionless face (see Figure 3). In the context of a social interaction a blank face can easily be interpreted as angry. Our explanation is that during the performance people do not consider themselves to be actively socially engaged and in this context a blank face is more plausibly interpreted as a signal of attention or concentration (Healey et al., 2017).

### 4.2. Head, Torso and Hands

**Figure 4:**
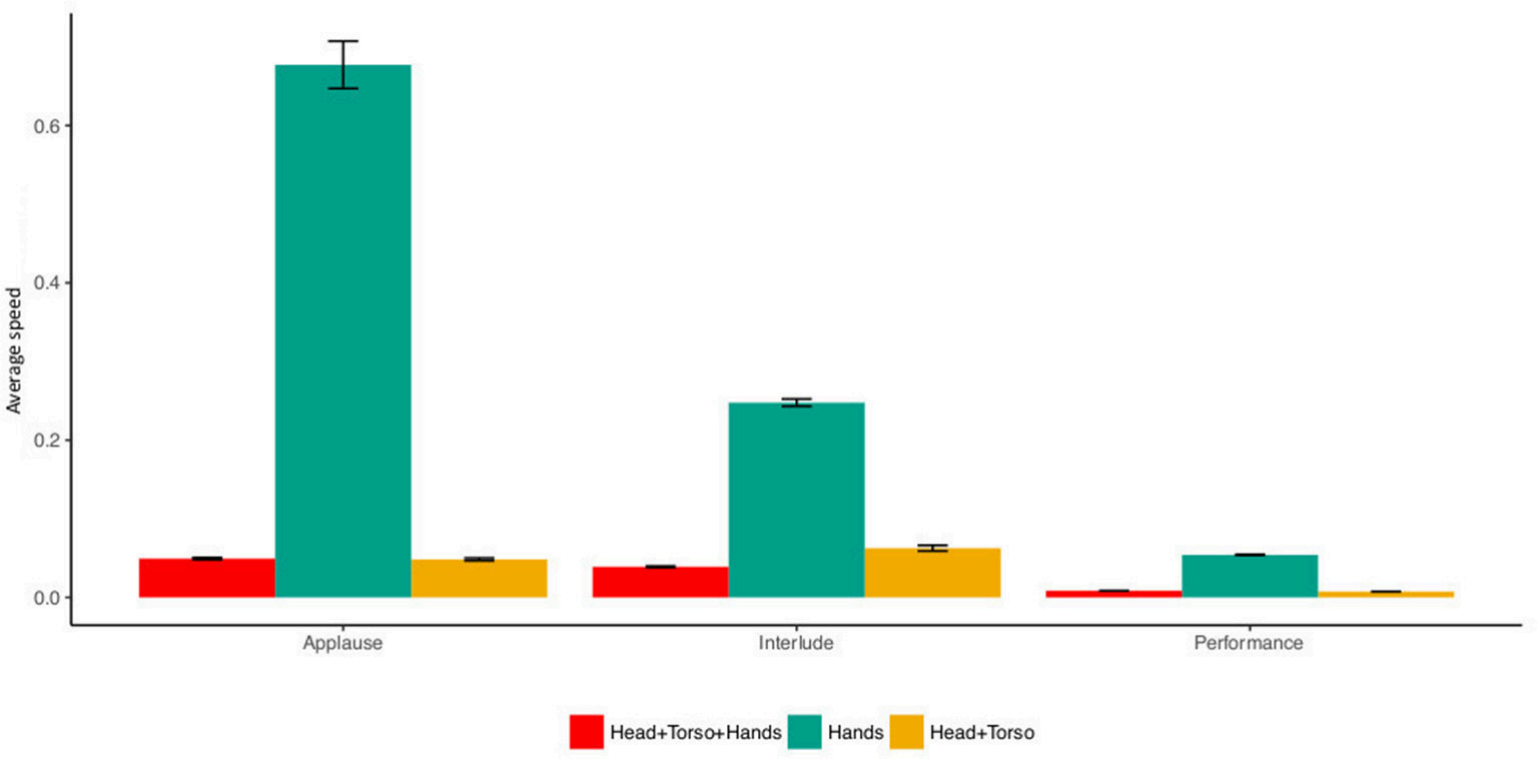
Bar plot of audience “Head, Torso and Hands,” “Hands,” and “Head and torso” in each part of the performance.

For audience upper body movement (head, torso and hands), we extracted data from 48 audience members (17 males) while for the “hands” and “head and torso” data, the sample size reduced to 38 audience members (11 males) since not all the participants wore the infrared wristband. The third line plot in Figure 3 shows the average upper body movement of the audience during the performance parts and the interludes. It is clear from the plot that the audience move much more during applause and interludes than during the performances. Figure 4 shows the breakdown of average movement by body parts: (1) Head, torso and hands, (2) Head and torso, and (3) Hands during different parts of the performance. It is apparent from the plot that the hands are consistently the most mobile part of the body and the most potentially salient during a performance. This supports (H1) and suggests that compared to the other parts of the body, hands are the best candidate for a movement response that is detectable by dancers. However, it was expected that average head, torso and hands to always be equal or higher to average head and torso. This is not visible in the interlude part of Figure 4. This is due to the erratic audience behavior that affected the efficiency of the tracking during the interludes we therefore exclude this from further analysis.

### 4.3. Survey I: Ranking the Performances

The survey results indicate that the 2nd performance is the most preferred (total ranking=50), 3rd was ranked second (total ranking = 54) while the 1st (total ranking = 57) and 4th (total ranking = 59) are the least preferred. This correlates with the overall movement of the audience with the 2nd (*M* = 0.0071, *SD* = 0.0032) and 3rd (*M* = 0.0078, *SD* = 0.0042) performances being the ones with least movement while in the 4th (*M* = 0.008, *SD* = 0.004) and 1st (*M* = 0.0101, *SD* = 0.0062) parts the audience tends to move more. Spearman's rank correlation suggests a high correlation between average audience movement and average ranking of the four performances (*r* = 0.8). However, the result is not statistically significant (*p* = 0.33) mainly due to the low sample size of the performances. Looking at the overall metrics in one performance compared to the other is a low powered way to identify moments of high or low engagement in the audience and cannot distinguish moment-by-moment changes in engagement or boredom.

### 4.4. Survey II: Assessing Audience Engagement From Movement

A GLMM with a linear model was used to test for differences in the engagement ratings for high and low movement clips. To do this, the movement state (moving vs. non-moving) and connection of participants to dance (performers vs. non-performers) were tested as fixed factors and the participant and number of times they attended a dance performance in a year (0–4) as random factors. The results show a main effect of the movement state on the engagement scores (Chi-sq = 95, *p* < 0.01), with participants rating the audience clips where the audience was moving less as the most engaged. The model does not show any effect of participants connection to dance (Chi-sq = 0.22, *p* = 0.63) on the engagement scores. Overall, this finding suggests that participants reported that audience members were more engaged to the performance when they were moving less. The GLMM results are reported in Table 3 below.

**Table 3:** GLMM model for engagement levels.

	**Estimate**	**Std. error**	**df**	***t*-value**
Movement state (moving)	1.02	0.11	298.00	9.73
Connection to dance (performer)	–0.13	0.29	11.00	–0.44

### 4.5. Granger Causality Analysis

**Figure 5:**
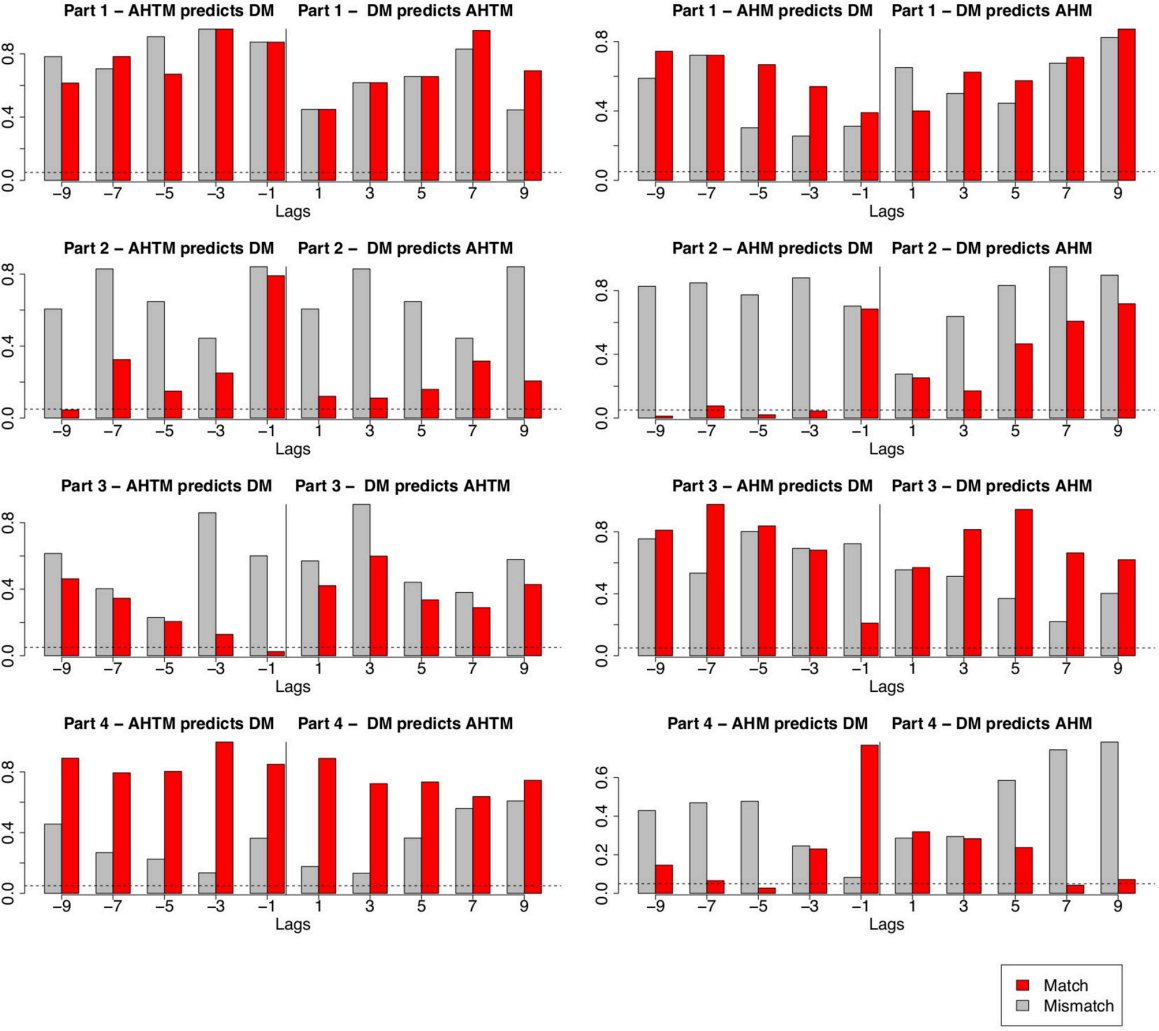
GC for audience head and torso movement (AHTM) and dancers movement (DM) in each performance part (plots in left column), GC for audience hand movement (AHM) and dancers movement (DM) in each performance part (plots in right column). The x axis indicates the lag order and the y axis the *p*-values. The dashed line indicates a significance level of *p* ≤ 0.05.

Since our final hypothesis focuses on kinesthesia (H3), we used Granger causality (GC) analysis to test if audience movement can be predicted from the movement of the dancers. GC accounts for the presence of autocorrelations and is able to identify meaningful lagged relationships between two timeseries at different timescales (Dean and Bailes, 2010). A predictor variable, x, is said to “Granger cause” a response variable y, if information about the previous values of x is useful in predicting future values of y, over and above prediction based on information about previous values of y alone (Dean and Dunsmuir, 2016). We examine this for each part of the performance separately and for lags between –9 and +9 seconds. Existing research suggests that there are several ways to identify the appropriate lag structure for the GS analysis. One way is to choose among a wide variety of model selection criteria. However, according to Thornton and Batten (1985) different selected statistical criteria for determining the lag structure might show contradictory conclusions on the GC results while it appears that the safest approach is to perform an extensive search of the lag space. Based on this, we chose the lag order based on the frequency of the data. Since the frequency of the data was 1 Hz, we decided to use as a starting point the lag order of 1 and test GS for lags twice the frequency. This is also supported by the research of Muth et al. (2015) and Vicary et al. (2017) that argues that the aesthetic responses to dynamic art forms such as dance and music are likely to involve a sampling period of at least a couple of seconds. Based on this, we assessed Granger causal relationships at temporal delays between 1 and 9 s. Positive lags indicate dancers movement predicting audience movement while negative lags indicate the opposite (Figure 5). We checked for causality separately between audience hand movement and dancers movements and between audience head and torso movement and dancers movements. To ensure stationarity, all time-series were differenced by subtracting consecutive sample points from each other prior to applying GC. We tested this both for matching responses (e.g., responses from the same performance part, audience body movement from Part2 with dancers movement from Part2) and for mismatching responses (e.g., responses from the different performance parts, audience body movement from Part2 with dancers movement from Part4). Randomly mismatching responses should cancel significant relationships between dancers and audiences that exist for responses that are derived from the same performance. Overall, the GC results show that dancers movements don't systematically predict audience movement. This is not consistent hypothesis 3. Moreover, the results show a systematic prediction in the opposite direction. In particular, we found that in Parts 2 and 3 dancers movement is predicted by the audience movement. The results are reported separately for each performance part as seen in the plots in Figure 5. There are no statistically significant GC relationships between either hand or head and torso movement and dancers movement for Part 1. For Part 2, audience hand movement predicts dancers movement at a lag order of 3 s, *F*_(3, 1120)_ = 2.71, *p* = 0.04, 5 s, *F*_(5, 1116)_ = 2.68, *p* = 0.02 and 9 s, *F*_(9, 1108)_ = 2.35, *p* = 0.01. Similarly in Part 2 audience head and torso movement predicts dancers movement at a lag order of 3 s, *F*_(3, 1108)_ = 1.92, *p* = 0.04. For part 3, audience head and torso movement predicts dancers movement at a lag order of 1 s, *F*_(1, 1119)_ = 5.03, *p* = 0.02 while for part 4 we found a bidirectional relationship. Audience hand movement predicts dancers movement at a 5 s lag order *F*_(5, 1208)_ = 2.53, *p* = 0.02 but also dancers movement predict audience hand movement at a lag order of 7 s, *F*_(7, 1204)_ = 2.08, *p* = 0.04. This suggests that in part 4 there should be another exogenous variable that influences both audience and dancers.

## 5. Discussion

The results of the study provide evidence that there is a systematic relationship between audience body movement and engagement, but also that the relationship is perhaps a surprising one. The first and most obvious point about the audience's visible responses is that they are much stronger during moments of applause and intervals than during a performance. As we found in previous studies (Theodorou et al., 2016; Theodorou and Healey, 2017) audiences move very little and have predominantly expressionless faces during the actual performance. This is in clear contrast to the animated facial expressions and body movements that are apparent during intervals.

One simple reason for this is that audiences are physically restricted during a performance and convention requires them to sit quietly on a chair observing and making sure they don't annoy the performers or the rest of the audience. However, this is not as trivial as it seems. People could equally well sit still with a smile on their faces or a look of rapture. We interpret the predominantly blank faces during the performance as indicating that people do not believe they are actively engaged in a social encounter during the performance. Blank faces predominate because of the absence of non-verbal responsiveness typical of social encounters, such as smiles, gestures and nods (Goodwin, 1979; Bavelas et al., 2000). The facial expressions and hand gestures of contemporary dance audiences are different from those involved in focused social interaction. Instead of the content specific gestures typical of conversation (McNeill, 2005) the hand movements of an audience during a performance appear to be primarily concerned with functional matters like scratching or adjusting glasses (Theodorou et al., 2016; Theodorou and Healey, 2017)

Although audience movements appear to be incidental, the results provide evidence that this class of audience body movements, especially their hand movements, may nonetheless provide important real-time signal of levels of engagement. Firstly the analysis shows that hand movements are the most frequent and potentially detectable movements for the dancers. Head and torso movements are less visually salient although it is possible that they might also be a significant component of audience response. Importantly, the hands have more degrees of freedom to move independently from the rest of the body. What hand movements are most likely to signal, we propose, is disengagement. As Galton (1885) and Pasquier (2015) observed for audiences in lectures and theaters, incidental increase of movement suggests that people are becoming restless. This is consistent with the literature that claims that more spontaneous self-touching gestures correlate with audience boredom or nervousness (Mahmoud and Robinson, 2011; Theodorou and Healey, 2017). This interpretation is supported by our main finding that independent judges rate audience engagement as highest in clips where they move least. Interestingly, judges level of familiarity with contemporary dance does not appear to affect this judgement. If we are correct, this leads to the conclusion that the most compelling real-time signal of audience engagement in contexts like contemporary dance are moments of *collective stillness*. These moments are visible and, in the right circumstances, audible for performers.

This claim may help to explain the results of the Granger causality analysis. During the moments when an audience becomes more engaged during a performance they should, by hypothesis, move less and we therefore don't expect a simple relationship between total amount of movement by dancers and audience members. As noted, the GC analysis does not show any systematic influence of movement on the stage on audience movement. While this is compatible with the idea of collective stillness it is not easily reconciled with the movement simulation hypotheses (Reason and Reynolds, 2007; Winters, 2008; Jola et al., 2011a) and contrasts with research on kinesthesia. The kinesthesia hypothesis normally focuses on the brain responses of participants and not on the overt body movement that is the main focus of this paper. Therefore, one line of response might be that kinesthesia is a covert experience that cannot be detected by external observation. If this is true it rules out kinesthestic responses as a direct basis for the signals that underpin audience-performance interaction. Alternatively, it could be that kinesthestic responses are in fact manifest in body movements but can only be measured using much more fine grained techniques than the computer vision approaches used here. While this preserves the potential for simluative or kinesthetic responses to contribute to performer-audience interaction it doesn't explain why these much more fine-grained movements would override the more obvious overt responses that can currently be tracked. A last possibility is that kinesthetic (or simulative) responses may have the effect of suppressing the incidental movements that, we argue, are key to understanding how performers can sense ongoing audience responses. This is an open question but we think it a more plausible explanation is that reduced incidental movement reflects increased attention and interest in the ongoing events.

Perhaps the most surprising finding presented here is the evidence that audience movement ‘Granger causes’ the movement of the dancers and not vice versa. According to Dean and Bailes (2010) research on real-time perception of music, listeners cannot influence acoustic parameters (such as the intensity or the spectral flatness of the audio) and these should be treated as exogenous or independent variables that can influence perceptual parameters (such as perceptions of change and expressed affect—arousal and valence—in music) which are considered endogenous or dependent variables. Could something similar happen in dance? One speculative possibility is that the choreography, as an exogenous variable, builds up specific expectations about what is happening which may affect the audience in advance of manifest events on stage. This is something that needs further investigation that focuses more on the aesthetics elements of a dance performance.

## 6. Conclusion

Audiences are fundamental for live performance in a wide range of contexts e.g.,: comedy, theater, dance, concerts, lectures. Our guiding intuition is that one of the defining features of these situations is the real-time interaction between performers and audiences. This naturally leads to questions about what patterns of audience response could actually be detected by performers on stage. While the interaction between audience and performers is especially explicit in genres such as stand-up comedy, our study suggests that it is much more subtle in genres like contemporary dance. The contrast between non-verbal behaviors during a performance and those between performances suggests that people's responses are strongly affected by whether they consider themselves to be engaged in a focused social encounter. In genres like street performance and stand-up comedy—and in the intervals between dance performances—people make active use of non-verbal cues such as facial expression. Our argument is that in contexts like contemporary dance the important real-time response cues during the performance are not gestures or facial displays but the incidental movements that can signal people's level of interest in a performance. Existing audience research has often focused on covert physiological responses or self reported measurements that may correlate with, but cannot account for the dynamics of live interaction among performers and audiences. This study provides evidence that during a live performance overt audience responses matter. We filmed the audiences and dancers in four contemporary dance performances and extracted body, hand and facial continuous data using computer vision techniques. Our results support the proposal that a key signal of audience engagement is collective stillness; moments that everyone in the room can sense. This explanation is consistent with the finding that there appears to be no systematic effect of dancers movements on audience movement. The significance of audience responses can only be properly understood, we argue, in the context of an analysis of that performance as a structured social encounter.

## Author Contributions

This work is based on research carried out for the Ph.D. by LT under the primary supervision of PH and secondary supervision of FS. LT and PH: concept, experimental design, and results interpretation; LT: data collection; LT and FS: signal processing; LT, PH, and FS: statistical analysis; LT: writing original draft; PH and FS: writing review and editing.

### Conflict of Interest Statement

The authors declare that the research was conducted in the absence of any commercial or financial relationships that could be construed as a potential conflict of interest.
